# IFN-γ modulates Ly-49 receptors on NK cells in IFN-γ-induced pregnancy failure

**DOI:** 10.1038/srep18159

**Published:** 2015-12-11

**Authors:** Zhong-Yin Li, Zhi-Hui Song, Chao-Yang Meng, Dan-Dan Yang, Ying Yang, Jing-Pian Peng

**Affiliations:** 1 State Key Laboratory of Stem Cell and Reproductive Biology, Institute of Zoology, Chinese Academy of Sciences, Beijing, P.R. China; 2 University of Chinese Academy of Sciences, Beijing, P.R. China

## Abstract

We have previously shown that interferon gamma (IFN-γ) induces aberrant CD49b^+^ natural killer (NK) cell recruitment by regulating CX3CL1 and eventually provokes foetal loss. In this study, we show that IFN-γ also modulates Ly-49 receptors on NK cells during pregnancy failure. The percentages of Ly-49A^+^ and Ly-49G2^+^ NK cells in the uteri of the IFN-γ-treated group were significantly lower than those observed in the control group. Moreover, the median fluorescence intensity (MFI) values of Ly-49A and Ly-49G2 expression on NK cells in the uteri of the IFN-γ-treated group were significantly lower than those of the control group. Using isolated spleen leucocytes, we further found that IFN-γ significantly reduced the percentage of Ly-49A^+^ NK cells *in vitro*. However, CX3CL1 was not involved in the modulation of Ly-49 receptors, and the expression of CX3CR1 was not regulated by IFN-γ in spleen leucocytes. Collectively, our data indicate that IFN-γ can modulate Ly-49 receptors on NK cells and this process may play a role in IFN-γ-induced pregnancy failure. Thus, we provide a new line of evidence correlating the deleterious effects of IFN-γ with its role in regulating NK cell Ly-49 receptors during pregnancy failure.

Natural killer (NK) cells, which lack characteristic B and T cell surface antigens, were initially referred to as null lymphocytes^1^. NK cells regulate their activation and effector functions by using a sophisticated repertoire of cell surface receptors^2^. The Ly-49 receptors, which are involved in NK cell recognition of polymorphic major histocompatibility complex (MHC) class I molecules, include receptors with either inhibitory or activating functions^3^,^4^. The mouse Ly-49 family encompasses more than 15 functional members^5^, and the repertoire of Ly-49 receptors are associated with different mouse strains^6^. BALB/c mice possess six inhibitory receptors (Ly-49A, B, C, E, G2, I) and a stimulatory receptor (Ly-49L)^4^,^6^.

In women, rodents, and pigs, uterine NK (uNK) cells have been transiently found within the uterine endometrium^7^. Peripheral NK (pNK) and uterine NK (uNK) cells have been associated with reproductive failure, although the prognostic value of measuring pNK or uNK cell parameters remains uncertain^8^. Previous studies have shown that women who have experienced recurrent spontaneous abortions have a limited repertoire of inhibitory receptors of the killer immunoglobulin-like receptor (KIR) family, which constitutes one of the MHC class I receptor families^9^. However, little data currently exist regarding the alterations of Ly-49 receptors during pregnancy failure.

The role of NK cells in the protection against pathogens and tumors includes cell cytotoxicity and the secretion of interferon gamma (IFN-γ)^10^. Despite numerous findings that have shown that IFN-γ secretion is essential for NK cell functional activity, little is known regarding whether IFN-γ regulates the Ly-49 receptors on NK cells.

Chemokines are small cytokines with selective chemoattractant properties that coordinate leucocyte trafficking^11^. Many chemokines have been shown to augment the cytolytic activity of NK cells by promoting cytotoxic granule release^12^. On the basis of the findings regarding the chemokine receptor CX3CR1, two killer lectin-like receptor G1 (KLRG1^+^) mouse NK cell subsets have been defined, and KLRG1^+^CX3CR1^+^ NK cells have been found to display impaired IFN-γ production and tumor cell lysis^13^. We have previously demonstrated the deleterious effects of IFN-γ on pregnancy via the aberrant regulation of CX3CL1 and CD49b^+^ NK cells^14^. Thus, whether the CX3CL1/CX3CR1 axis is also involved in the regulation of NK cell activity during pregnancy failure is an interesting question to answer.

The aim of the present study was to investigate whether IFN-γ-induced pregnancy failure is associated with the alterations of Ly-49 receptors and whether IFN-γ and CX3CL1 modulate the expression of Ly-49 receptors. Here, we report that IFN-γ-induced pregnancy failure correlates with Ly-49A and Ly-49G2, and further mechanisms of action are discussed. Our results suggest the prognostic value of Ly-49 receptors in reproductive failure.

## Results

### IFN-γ altered the expression of Ly-49 receptors on NK cells in IFN-γ-induced pregnancy failure *in vivo*

**Figure 1:**
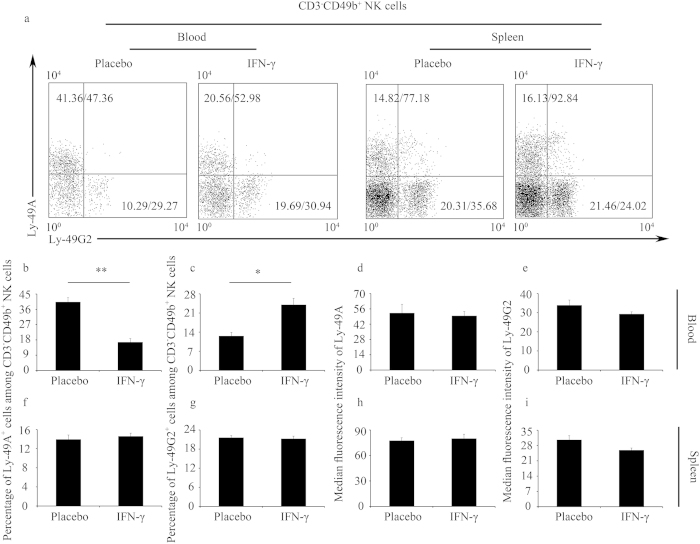
IFN-γ altered the percentages of Ly-49^+^ NK cells in the blood in an IFN-γ-induced pregnancy failure model. (**a–i**) Syngeneically mated BALB/c females were injected with placebo or IFN-γ intraperitoneally on GD6 and sacrificed on GD8. (**a**) Representative flow cytometric analysis of Ly-49A and Ly-49G2 expression on gated CD3^−^CD49b^+^ NK cells in the blood and spleen. See Supplementary Fig. S1b, c for the gating strategy; the numbers in the dot plots indicate the percentages of the Ly-49^+^ NK cells (gated on CD3^−^CD49b^+^ NK)/MFI values of Ly-49 receptor expression. Data summary of the percentages of Ly-49^+^ NK cells (**b,c,f,g**) and MFI values of Ly-49 receptor expression (**d,e,h,i**) in the blood (**b–e**) and spleen (**f–i**). Data show the mean ± SEM of three (blood) or four (spleen) independent experiments and were obtained from three (blood) or four (spleen) mice per group, respectively. ^*^P < 0.05, ^**^P < 0.01 by independent sample T-test.

Our previous study has shown that IFN-γ treatment enhanced the accumulation of the CD49b^+^ NK cell subset in the uterus and blood^14^. Furthermore, the cytotoxicity of CD49b^+^ NK cells is higher than that of CD49b^−^ NK cells^15^. Thus, we examined whether IFN-γ regulated NK cell receptors in addition to NK cell recruitment during IFN-γ-induced pregnancy failure. For this purpose, we tested whether IFN-γ modulated the expression ***in vivo*** of the Ly-49 family inhibitory receptors, which play a critical role in controlling the immune functions of NK cells^4^. Using an IFN-γ-induced abortion mouse model as previously described^14^, we detected a significantly higher level of IFN-γ in the blood of IFN-γ-treated mice (Supplementary Fig. S1a), suggesting that IFN-γ is a mediator of pregnancy failure. The percentage of Ly-49A^+^ cells among CD3^−^CD49b^+^ NK cells in the blood (see Supplementary Fig. S1b for the gating strategy) from the IFN-γ-treated group was significantly lower than that in the control group (Fig. 1a,b). In contrast, the percentage of Ly-49G2^+^ cells among CD3^−^CD49b^+^ NK cells in the blood (see Supplementary Fig. S1b for the gating strategy) from the IFN-γ-treated group was significantly higher than that in the control group (Fig. 1a,c). However, the median fluorescence intensity (MFI) values of Ly-49A and Ly-49G2 expression in the blood were similar between the two groups (Fig. 1a,d,e). Moreover, we assessed whether similar results were observed in the spleen. Nevertheless, the percentages of Ly-49A^+^ and Ly-49G2^+^ cells among CD3^−^CD49b^+^ NK cells (see Supplementary Fig. S1c for the gating strategy) and the MFI values of Ly-49A and Ly-49G2 expression in the spleen were similar between the IFN-γ-treated and the control groups (Fig. 1a,f–i).

**Figure 2:**
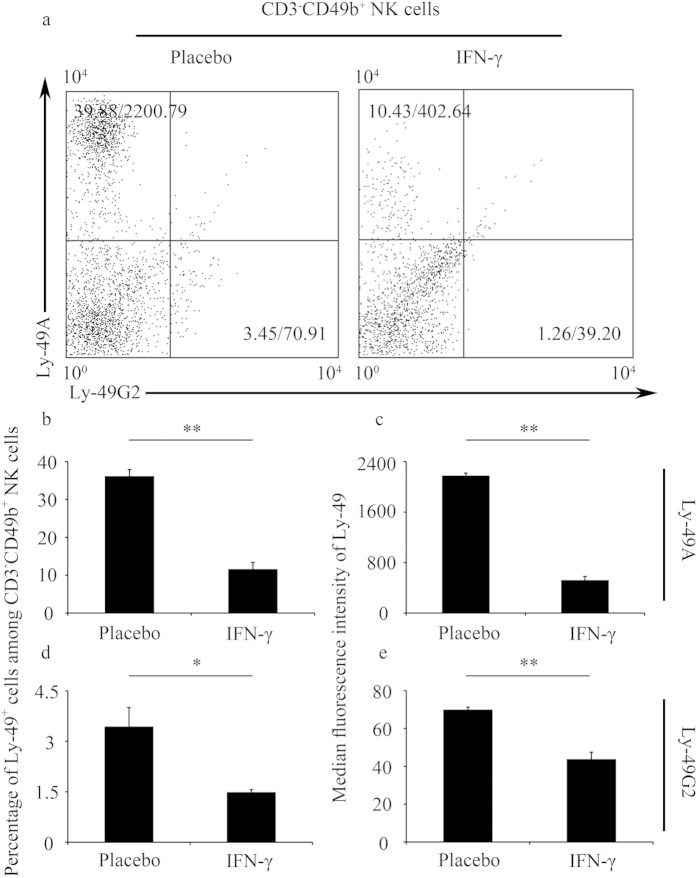
IFN-γ altered the expression of Ly-49 receptors on NK cells in the uterus in an IFN-γ-induced pregnancy failure model. (**a–e**) Syngeneically mated BALB/c females were injected with placebo or IFN-γ intraperitoneally on GD6 and sacrificed on GD8. (**a**) Representative flow cytometric analysis of Ly-49A and Ly-49G2 expression on gated CD3^−^CD49b^+^ NK cells in the uterus. See Supplementary Fig. S2 for the gating strategy; the numbers in the dot plots indicate the percentages of the Ly-49^+^ NK cells (gated on CD3^−^CD49b^+^ NK)/MFI values of Ly-49 receptor expression. Data summary of the percentages of Ly-49^+^ NK cells (**b,d**) and MFI values of Ly-49 receptor expression (**c,e**). Data show the mean ± SEM of three independent experiments and were obtained from three mice per group. ^*^P < 0.05, ^**^P < 0.01 by independent sample T-test.

Furthermore, uteri were harvested to assess the expression of Ly-49 receptors on NK cells. Ly-49A was expressed at a higher level in the uterus compared with the blood and spleen (Fig. 1a and Fig. 2a), indicating a potential role for Ly-49 receptors in forming a specialized immune milieu at the maternal-fetal interface. The percentages of Ly-49A^+^ and Ly-49G2^+^ cells among the CD3^−^CD49b^+^ NK cells in the uterus (see Supplementary Fig. S2 for the gating strategy) in the IFN-γ-treated group were significantly lower than those in the control group (Fig. 2a,b,d). In addition, the Ly-49A and Ly-49G2 expression MFI values in the IFN-γ-treated group were significantly lower than those in the control group (Fig. 2a,c,e). Thus, further experiments were designed to explore the causes underlying IFN-γ-induced alterations of Ly-49 receptor expression.

### IFN-γ but not CX3CL1 modulated the percentages of Ly-49^+^ NK cells *in vitro*

**Figure 3:**
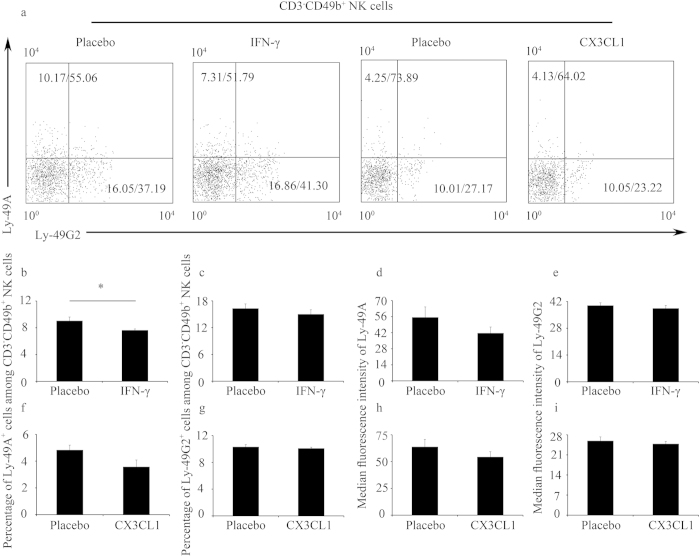
IFN-γ but not CX3CL1 modulated the percentages of Ly-49^+^ NK cells *in vitro*. (**a–i**) Splenic leucocytes were cultured with IFN-γ (250 U/ml) or CX3CL1 (250 ng/ml) for 24 h. (**a**) Representative flow cytometric analysis of Ly-49A and Ly-49G2 expression on gated CD3^−^CD49b^+^ NK cells; the numbers in the dot plots indicate the percentages of Ly-49^+^ NK cells (gated on CD3^−^CD49b^+^ NK)/MFI values of Ly-49 receptor expression. Data summary of the percentages of Ly-49^+^ NK cells (**b,c,f,g**) and MFI values of Ly-49 receptor expression (**d,e,h,i**) after IFN-γ (**b–e**) and CX3CL1 (**f–i**) treatment. Data show the mean ± SEM of four (IFN-γ) or three (CX3CL1) independent experiments, respectively. ^*^P < 0.05, by independent sample T-test.

To investigate how IFN-γ modulates the expression of Ly-49 receptors, we performed spleen leucocyte culture experiments ***in vitro***. We first found that IFN-γ treatment did not alter the percentage of CD3^−^CD49b^+^ NK cells *in vitro* (Supplementary Fig. S3a, b). However, IFN-γ significantly reduced the percentage of Ly-49A^+^ cells among CD3^−^CD49b^+^ NK cells (Fig. 3a,b). In contrast, IFN-γ did not alter the percentage of Ly-49G2^+^ cells among CD3^−^CD49b^+^ NK cells (Fig. 3a,c). Similarly, IFN-γ treatment had no significant effect on the MFI values of Ly-49A and Ly-49G2 expression (Fig. 3a,d,e).

**Figure 4:**
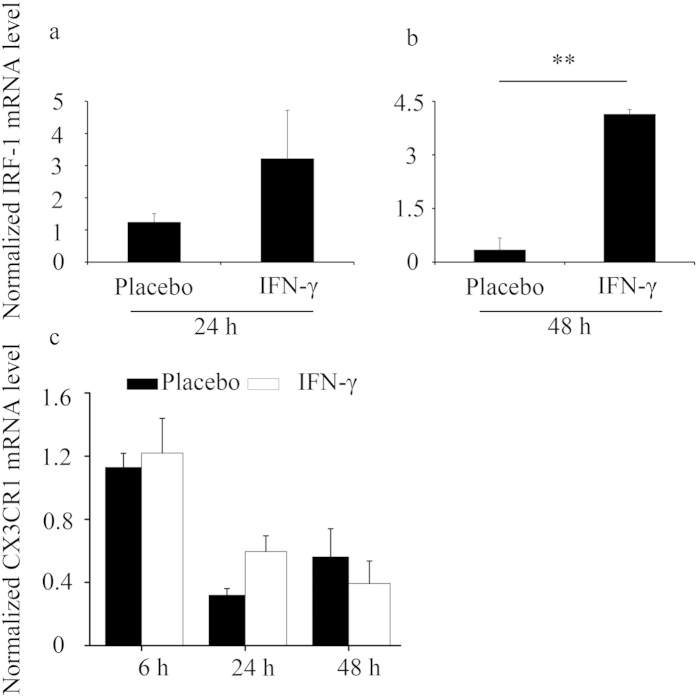
CX3CR1 mRNA levels were not regulated by IFN-γ in splenic leucocytes. (**a–c**) Splenic leucocytes were cultured with IFN-γ at a dose of 250 U/ml. At the indicated times, cells were harvested to determine IRF-1 (**a,b**) and CX3CR1 (**c**) expression by quantitative PCR. Data show the mean ± SEM of four independent experiments. ^**^P < 0.01 by independent sample T-test.

Furthermore, we determined whether the alteration of Ly-49A^+^ cell percentage was a secondary indirect event caused by IFN-γ. We have previously shown that IFN-γ significantly upregulates CX3CL1 expression^14^, and CX3CL1 exhibits an efficient chemotactic activity for NK cells^16^. Thus, we examined whether CX3CL1 altered Ly-49 receptor expression. As shown in Supplementary Fig. S3a, c, CX3CL1 did not alter the percentage of CD3^−^CD49b^+^ NK cells *in vitro* as IFN-γ did (Supplementary Fig. S3a, b). In addition, CX3CL1 had no significant effects on both the percentages of Ly-49A^+^ and Ly-49G2^+^ cells and the MFI values of Ly-49A and Ly-49G2 expression *in vitro* (Fig. 3a,f–i). Moreover, we detected that CX3CR1 mRNA levels were not regulated by IFN-γ at the indicated times in spleen leucocytes (Fig. 4c). As a positive control, IRF-1 was greatly induced by IFN-γ (Fig. 4a,b), which is consistent with previous data^17^. Collectively, these ***in vitro*** results suggest that the alteration of Ly-49 receptor expression is not dependent on CX3CL1.

### CXCL12 did not modulate the percentages of Ly-49^+^ NK cells *in vitro*

**Figure 5:**
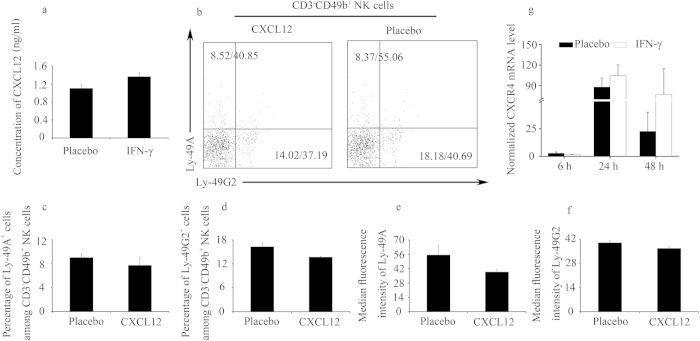
CXCL12 did not modulate the percentages of Ly-49^+^ NK cells *in vitro*. (**a**) Syngeneically mated BALB/c females were injected with placebo or IFN-γ intraperitoneally on GD6 and sacrificed on GD8. CXCL12 concentration in the serum was determined by ELISA. Data show the mean ± SEM of four independent experiments and were obtained from four mice per group. (**b–f**) Splenic leucocytes were cultured with CXCL12 at a dose of 500 ng/ml for 24 h. (**b**) Representative flow cytometric analysis of Ly-49A and Ly-49G2 expression on gated CD3^−^CD49b^+^ NK cells; the numbers in the dot plots indicate the percentages of the Ly-49^+^ NK cells (gated on CD3^−^CD49b^+^ NK)/MFI values of Ly-49 receptors expression. Data summary of the percentages of Ly-49^+^ NK cells (**c,d**) and MFI values of Ly-49 receptor expression (**e,f**). Data show the mean ± SEM of three independent experiments. (**g**) Splenic leucocytes were cultured with IFN-γ at a dose of 250 U/ml and at the indicated times, splenic leucocytes were harvested to determine CXCR4 expression by quantitative PCR. Data show the mean ± SEM of four independent experiments.

CXCL12 and its receptor CXCR4 have been shown to be involved in the migration of NK cells^18^. Beyond the control of leucocyte trafficking, the CXCL12/CXCR4 axis has also been shown to play crucial roles during zebrafish and mouse embryonic development^19^. Significantly upregulated CXCL12 and CXCR4 expression was detected in the uteri of IFN-γ-induced abortion mice ***in vivo***, and CXCL12 facilitated peripheral CD49b^+^ NK cell migration ***in vitro*** (data not shown), suggesting an association between CXCL12 and IFN-γ-induced abortion. However, IFN-γ treatment did not lead to a similar enhancement of CXCL12 in the blood (Fig. 5a). Furthermore, we explored whether CXCL12 was involved in regulating the expression of Ly-49 receptors ***in vitro***. CXCL12 did not alter the percentage of CD3^−^CD49b^+^ NK cells (Supplementary Fig. S3a,d), which was comparable to the percentage observed when the cells were treated with CX3CL1 and IFN-γ (Supplementary Fig. S3a–c). Moreover, CXCL12 had no significant effects on both the percentages of Ly-49A^+^ and Ly-49G2^+^ cells and the Ly-49A and Ly-49G2 expression MFI values (Fig. 5b–f). Finally, we detected that CXCR4 was not induced by IFN-γ at the indicated times in the spleen leucocytes (Fig. 5g). Overall, our findings suggest that although CXCL12 correlates with IFN-γ-induced abortion, the alteration in Ly-49 receptor expression is not dependent on CXCL12.

## Discussion

Many human pregnancy complications have been shown to be associated with IFN-γ^20^. Our previous data have indicated that exogenous IFN-γ administration leads to the aberrant CD49b^+^ NK cell recruitment in a mouse model of fetal loss^14^. Here, we show that IFN-γ also modulates Ly-49 receptors on NK cells during pregnancy failure ***in vivo***. To our knowledge, this is the first evidence showing that the deleterious effects of IFN-γ correlate with its role in regulating NK cell Ly-49 receptors during pregnancy ***in vivo***.

NK cell effector functions are regulated by the integrated signals transduced by activating and inhibitory cell surface receptors^2^. Ly-49 receptors are type II C-type lectin-like glycoproteins and regulate NK cell development and function via binding to MHC class I and MHC class I-like proteins^4^. The Ly-49 receptor pattern on NK cells is thought to be involved in the generation of a self-tolerant, yet functional, NK cell repertoire^21^. In the ***in vivo*** assays of this report, both the percentages of Ly-49^+^ NK cells and the MFI values of Ly-49 receptors expression in the uterus of the IFN-γ-treated group were significantly lower than those of the control group, suggesting that NK cells may contribute to maternal immune tolerance to a semi-allogenic conceptus through Ly-49 receptors. Ly-49A was expressed at a higher level, whereas Ly-49G2^+^ NK cells accounted for a smaller proportion in the uterus compared with those observed in the blood and spleen. Ly-49A and Ly-49G2 are both inhibitory receptors in the BALB/c mice^4^, which suggests that different Ly-49 inhibitory receptors have varied effects during pregnancy.

A previous work has demonstrated that MHC I molecules modulate the Ly-49 receptor expression in at least two different ways: alteration of the numbers of cells expressing a given receptor and modulation of the levels of a given receptor at the cell surface^21^. Our ***in vivo*** results also showed alterations of the numbers of cells expressing Ly-49A and Ly-49G2, as well as the modulation of the levels of these receptors.

Our previous work has shown that IFN-γ markedly upregulates CX3CL1 expression^14^. Furthermore, CX3CL1 has been shown to modestly enhance NK cell cytotoxicity towards NK-sensitive target cells^12^. However, this work showed that CX3CL1 did not alter both the percentages of Ly-49^+^ NK cells and the MFI values of Ly-49 receptors ***in vitro*** assays. Moreover, CX3CR1 was not regulated by IFN-γ, and CX3CL1 expression was not detected in the spleen leucocytes treated either with or without IFN-γ ***in vitro*** assays (data not shown). Thus, our observations suggest that CX3CL1 is not involved in the modulation of Ly-49 receptors.

In summary, our data indicate that IFN-γ can modulate NK cell Ly-49 receptors, which may play a role in IFN-γ-induced pregnancy failure. These results provide a theoretical basis for these molecules as prognostic markers of human embryo abortion.

## Materials and Methods

### Mice

Adult inbred BALB/c mice were purchased from Vital River Laboratories (VRL, Beijing, China). Females were mated with males of the same strain at a 2:1 ratio, and the day when a copulatory plug was observed was termed gestational day (GD) 1. Mouse studies were approved by the Institutional Animal Care and Use Committee of the Institute of Zoology, Chinese Academy of Sciences. All animal procedures were conducted in accordance with Institutional Animal Care and Use Committee guidelines. IFN-γ (R&D Systems, Minneapolis, MN, USA) or placebo (PBS containing 0.1% BSA) was injected intraperitoneally as previously described^14^.

### Isolation and primary culture of splenic leucocytes

Splenic leucocytes from non-pregnant BALB/c mice were obtained by mechanical disruption using a 37-μm cell strainer with a rubber syringe plunger in RPMI-1640 medium (HyClone, Logan, UT, USA) supplemented with 1% fetal bovine serum (FBS; HyClone) and penicillin/streptomycin (100 U/ml). Red blood cells (RBCs) were lysed with ammonium chloride lysing solution (0.14 M NH_4_Cl, 10 nM KHCO_3_ and 1 nM EDTA). The cells were seeded at a density of 5 × 10^6^ cells/ml in a dish containing RPMI-1640 medium supplemented with 10% FBS and penicillin/streptomycin (100 U/ml) and then were cultured with IFN-γ (250 U/ml), CX3CL1 (250 ng/ml; R&D Systems) or CXCL12 (500 ng/ml; R&D Systems). At the indicated times, splenic leucocytes were harvested for staining or RNA isolation.

### Flow cytometry analysis

Peripheral blood was collected in heparin-treated disposable vacuum blood collection tubes. BALB/c spleen cell suspensions were prepared by gentle homogenization in RPMI-1640 medium supplemented with 1% FBS and then sifted through a 37-μm cell strainer. After RBCs were removed, the cells were resuspended in PBS containing 0.2% BSA for further staining.

Uteri were dissected free from the mesometrium, minced into small fragments and then placed in HBSS containing 200 U/ml hyaluronidase (Sigma-Aldrich, St. Louis, MO, USA), 1 mg/ml collagenase type IV (Sigma-Aldrich), and 0.2 mg/ml DNase (Sigma-Aldrich) for 20 min at 37 °C as previously described, with several modifications^22^. After the digestion, the cells were washed with PBS containing 0.2% BSA, incubated in the same buffer for 15 min at 37 °C, and then sifted through a 37-μm nylon mesh. After centrifugation, the cells were resuspended in PBS containing 0.2% BSA for further staining.

Cell viability was assessed by dye exclusion using trypan blue, and the viability was measured at least at 90% in each experiment (data not shown). Negative and single colour staining were used to set gating in the flow cytometry experiments (data not shown).

Cells suspensions were blocked with anti-mouse CD16/CD32 monoclonal antibody (mAb; clone 93; eBioscience, San Diego, CA, USA) and then the following fluorochrome-conjugated mAbs were used for quadruple staining: allophycocyanin (APC)-conjugated anti-CD49b (clone DX5; eBioscience), peridinin chlorophyll protein (PerCP) Cyanine5.5-conjugated anti-CD3 (clone 145-2C11; eBioscience), PerCP Cyanine5.5-conjugated anti-CD45 (clone 30-F11; eBioscience), fluorescein isothiocyanate (FITC)-conjugated anti-Ly-49G2 (clone 4D11; eBioscience) and phycoerythrin (PE)-conjugated anti-Ly-49A (clone A1; eBioscience). After staining, the cells were rinsed with PBS containing 0.2% BSA and analysed with a FACScalibur (BD Biosciences, Franklin Lakes, NJ, USA).

### Total RNA isolation and quantitative PCR

Total RNA was isolated with a kit (BioTeke, Beijing, China), and reverse transcriptase (Promega, Madison, WI, USA) was used to generate cDNA. Quantitative PCR was conducted using SYBR Green MasterMix (ComWin Biotech Co. Ltd, Beijing, China) according to the manufacturer’s instructions. The following primers were used in this study: CXCR4 (forward 5′-GAAGTGGGGT CTGGAGACTAT-3′; reverse 5′-TTGCCGACTATGCCAGTCAAG-3′), CX3CR1 (PrimerBank ID 31542434a1; forward 5′-GAGTATGACGATTCTGCTGAGG-3′; reverse 5′-CAGACCGAACGTGAAGACGAG-3′), glyceraldehyde-3-phosphate dehydrogenase (GAPDH; PrimerBank ID 126012538c2; forward 5′-TGACCTCAACTACATGGTCTACA-3′; reverse 5′-CTTCCCATTCTCGGCCT TG-3′), and IRF-1 (PrimerBank ID 6680467a1; forward 5′-ATGCCAAT CACTCGAATGCG-3′; reverse 5′-TTGTATCGGCCTGTGTGAATG-3′). The target gene mRNA expression was normalized to GAPDH expression and the fold change was calculated as 2 ^–△△Ct^ (cycle threshold).

### Enzyme-linked immunosorbent assay (ELISA) assays

Serum was prepared and stored at −20 °C before use. IFN-γ (eBioscience) and CXCL12 (R&D Systems) concentrations in the serum were determined by ELISA according to the manufacturer’s instructions. Samples were quantified against standard curves and optical densities were measured by an ELISA plate reader at 450-nm wavelength.

### Statistics

Differences were considered significant when P < 0.05. All statistical analyses were performed using SPSS version 16.0 (SPSS, Chicago, IL, USA).

## Additional Information

**How to cite this article**: Li, Z.-Y. *et al.* IFN-γ modulates Ly-49 receptors on NK cells in IFN-γ-induced pregnancy failure. *Sci. Rep.* **5**, 18159; doi: 10.1038/srep18159 (2015).

## Supplementary Material

Supplementary Information
