# Supplementary material for: IFN-γ modulates Ly-49 receptors on NK cells in IFN-γ-induced pregnancy failure
Source: Sci Rep. 2015 Dec 11;5:18159. doi: 10.1038/srep18159 (PMC4676035; doi:10.1038/srep18159)
Supplement: Supplementary Information [file srep18159-s1.pdf]

Title

IFN-γ modulates Ly-49 receptors on NK cells in IFN-γ-induced pregnancy failure

Authors

Zhong-Yin Li,[1] Zhi-Hui Song,[1,2] Chao-Yang Meng,[1,2] Dan-Dan Yang,[1,2] Ying Yang,[1] and Jing-Pian Peng[*,1]

Affiliations

[1]State Key Laboratory of Stem Cell and Reproductive Biology, Institute of Zoology, Chinese Academy of Sciences, Beijing, P.R. China; and [2]University of Chinese Academy of Sciences, Beijing, P.R. China

[*]Corresponding author: State Key Laboratory of Stem Cell and Reproductive Biology, Institute of Zoology, Chinese Academy of Sciences, Beijing, P.R. China. Tel: 86-10-64807183; Fax: 86-10-64807099; E-mail address: pengjp@ioz.ac.cn

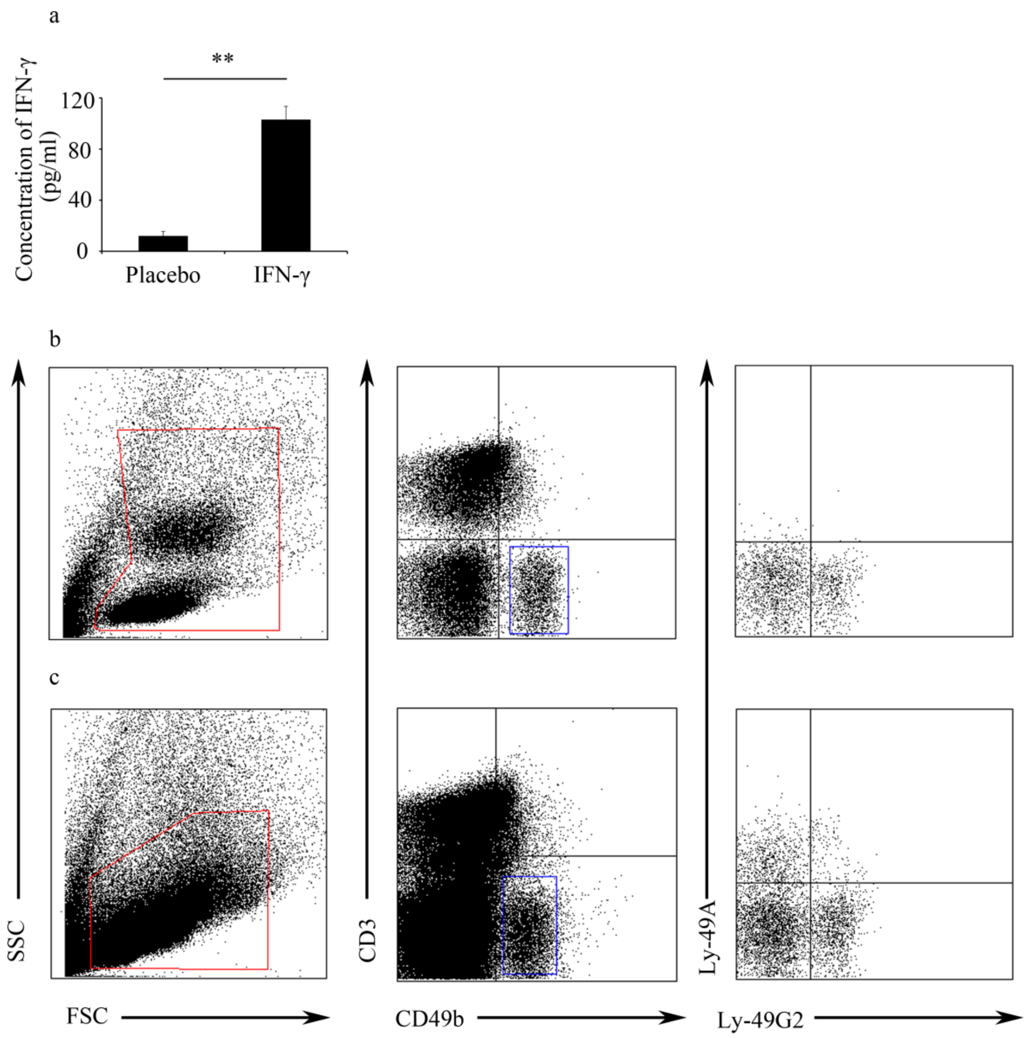

Supplementary Figure S1. The gating strategies to analyze the expression of Ly-49 receptors in the blood and spleen. (a) Syngeneically mated BALB/c females were injected with placebo or IFN-γ intraperitoneally on GD6 and sacrificed on GD8. IFN-γ concentration in the serum was determined by ELISA. Data show the mean ± SEM of three independent experiments and are obtained from three mice of each group. $^{**}P<0.01$ by independent samples T-test. (b) Dot plots shown are gating strategy to analyze the expression of Ly-49 receptors in the blood. Pan leucocytes are gated using FSC versus SSC (*left panel*) and NK cells are defined as $CD3^{-}CD49b^{+}$ (*middle panel*). Then, the expression of Ly-49 receptors is analyzed on $CD3^{-}CD49b^{+}$

NK cells (*right panel*). (c) Dot plots shown are gating strategy to analyze the expression of Ly-49 receptors in the spleen. Pan leucocytes are gated using FSC versus SSC (*left panel*) and NK cells are defined as CD3$^-$CD49b$^+$ (*middle panel*). Then, the expression of Ly-49 receptors is analyzed on CD3$^-$CD49b$^+$ NK cells (*right panel*). FSC, forward scatter; SSC, side scatter.

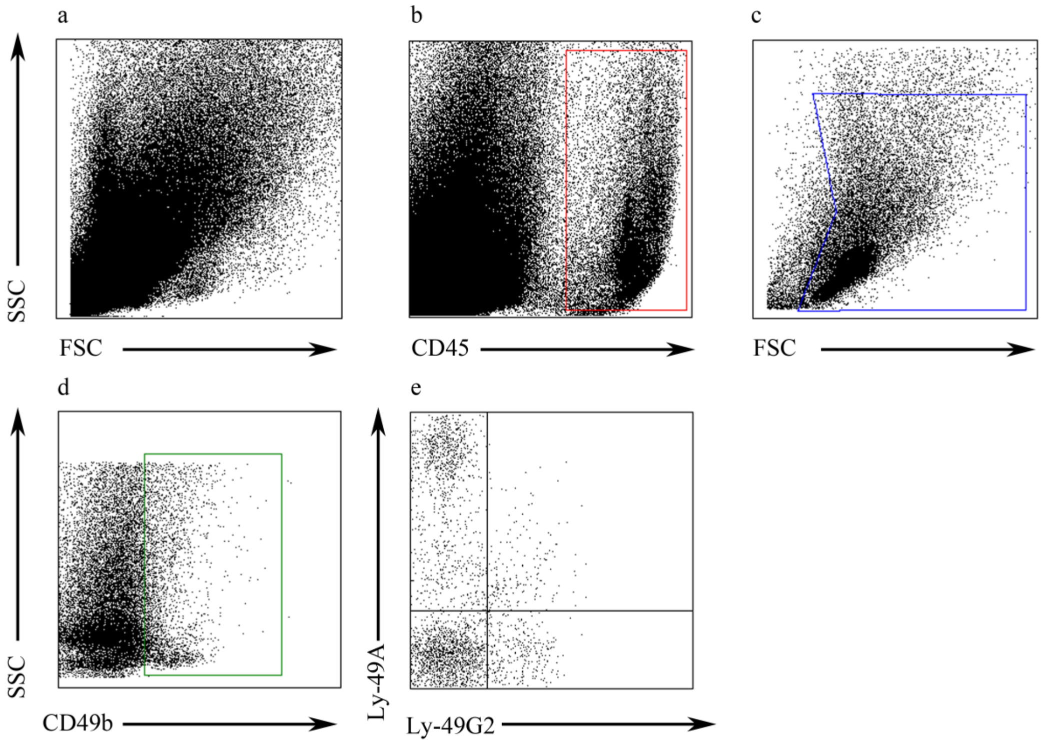

Supplementary Figure S2. The gating strategy to analyze the expression of Ly-49 receptors in the uterus. (a) Total cells are visualized using FSC versus SSC. Pan leucocytes are gated using anti-CD45 antibody versus SSC (b) and then back gate analysis of NK cells (c, d) is shown. NK cells are defined as CD45$^+$CD49b$^+$. Then, the expression of Ly-49 receptors is analyzed on CD45$^+$CD49b$^+$ NK cells (e). FSC, forward scatter; SSC, side scatter.

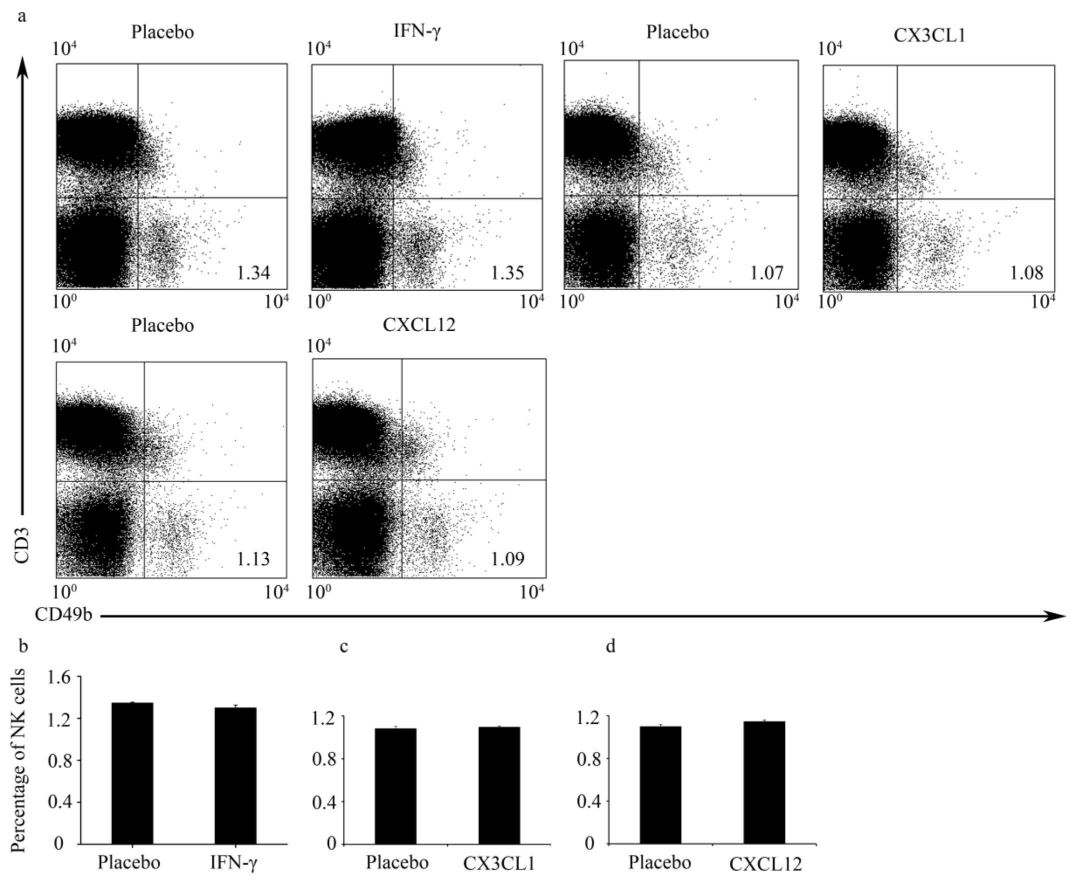

Supplementary Figure S3. IFN-γ, CX3CL1 and CXCL12 did not alter the percentage of $CD3^{-}CD49b^{+}$ NK cells in vitro. (a-d) Splenic leukocytes were prepared as described in Materials and Methods and then cultured with IFN-γ (250 U/ml) or CX3CL1 (250 ng/ml) or CXCL12 (500 ng/ml) for 24 h. (a) Representative flow cytometric analysis of the percentages of $CD3^{-}CD49b^{+}$ NK cells (lower-right quadrant) and numbers in dot plots indicate the percentages of $CD3^{-}CD49b^{+}$ NK cells. Data summary of the percentages of $CD3^{-}CD49b^{+}$ NK cells with IFN-γ (b), CX3CL1 (c) and CXCL12 (d) treatment. Data show the mean ± SEM of four (IFN-γ), three (CX3CL1) or three (CXCL12) independent experiments, respectively.
